# Immunity to Malaria in *Plasmodium vivax* Infection: A Study in Central China

**DOI:** 10.1371/journal.pone.0045971

**Published:** 2012-09-25

**Authors:** Kulachart Jangpatarapongsa, Hui Xia, Qiang Fang, Kaiming Hu, Yuanying Yuan, Meiyu Peng, Qi Gao, Jetsumon Sattabongkot, Liwang Cui, Baiqing Li, Rachanee Udomsangpetch

**Affiliations:** 1 Center for Innovation Development and Technology Transfer, Faculty of Medical Technology, Mahidol University, Bangkok, Thailand; 2 Department of Clinical Microbiology and Applied Technology, Faculty of Medical Technology, Mahidol University, Bangkok, Thailand; 3 Department of Parasitology, Bengbu Medical College, Anhui, China; 4 Anhui Key Laboratory of Infection and Immunity at Bengbu Medical College, Anhui, China; 5 Department of Immunology, Bengbu Medical College, Anhui, China; 6 Jiangsu Institute of Parasitic Disease, Wuxi, China; 7 Mahidol Vivax Research Center, Faculty of Tropical Medicine, Mahidol University, Bangkok, Thailand; 8 Department of Entomology, Pennsylvania State University, State College, Pennsylvania, United States of America; 9 Department of Pathobiology, Faculty of Science, Mahidol University, Bangkok, Thailand; 10 Center of Neglected Infectious Diseases, Mahidol University, Bangkok, Thailand; Universidade Federal de Minas Gerais, Brazil

## Abstract

**Background:**

*P. vivax* infection is characterised by relapsing fever, indicating reinfection by previously hidden parasites in the host. Relapsed infection can lead to the activation of the memory T cell pool, which may lead to protective immunity. This study aims to characterise immune responses in acute *P. vivax*-infected patients living in an area of central China characterised by only *P. vivax* infection.

**Methodology/Principal Findings:**

We conducted a cross-sectional immune-phenotypic analysis of adults using the following inclusion criteria: acute *P. vivax* infection (N = 37), a history of *P. vivax* infection (N = 17), and no known history of *P. vivax* infection (N = 21). We also conducted a 2-week longitudinal analysis following acute *P. vivax* infection, in which PBMC proliferation was measured in response to *P. vivax* and *P. falciparum* blood stage lysates. Using flow cytometry, we showed elevated memory T cells in the blood during acute *P. vivax* infection. The levels of γδ T cells were two-fold higher than those measured in naive controls. This result suggested that in the two populations, memory and γδ T cells promptly responded to *P. vivax* parasites. Interestingly, *P. falciparum* antigens stimulated T cells obtained from *P. vivax*-infected patients during a day 14-convalescence, whereas lymphocytes from the naïve control group responded to a lower degree of convalescence.

**Conclusions/Significance:**

Cell-mediated immunity during the convalescent period of the *P. vivax*-infected hosts was comprised of T cells that were specifically able to recognise *P. falciparum* antigens. Although the magnitude of the response was only half that measured after stimulation with *P. vivax* antigens, the matter of cross-antigenic stimulation is of great interest.

## Introduction

Malaria is a common tropical disease associated with mortality among children infected with *Plasmodium falciparum*, primarily in African countries [1]. In contrast, *P. vivax* causes relapsing infection and is rarely fatal in most areas to which it is endemic [2]. The underlying factors leading to this opposite outcome between the two malarial infections are still unclear. The unique host-parasite relationship and the immune response to *P. vivax* have not yet been elucidated. One reason for this lack of understanding is the difficulty in maintaining *P. vivax* in an *in vitro* culture [3], [4]. Therefore, the supply of *P. vivax* and progress in the relevant research is limited. Recent incidences of *P. vivax* infection have increased gradually in tropical countries [5]. Whether this increased incidence indicates slow development of drug resistance by the *P. vivax* parasite remains to be verified. Several studies have been conducted in the regions in which *P. falciparum* is present along with *P. vivax*. These studies have shown that immunity to *P. falciparum* parasites readily exists in the host [6], [7], [8], [9], [10], [11], [12]. Thus, the majority of studies analysing immunological parameters and malaria severity have been performed in the regions where *P. falciparum* and *P. vivax* co-infection is normally found [13], [14], [15], [16], [17], [18], [19]. Recent studies suggest that immune suppression occurs in response to *P. vivax* infection because of elevated regulatory T cell levels [20], [21]. The strain-specific and serological cross-reactive immunity between blood stage antigens of *P. falciparum* and *P. vivax* has been well-documented [12], [22], [23]. One report has shown no effect on the cell-mediated response [24]. To verify immunity to malaria in *P. vivax* infection without an interfering immune response caused by *P. falciparum*, we chose to focus on central China, where *P. vivax* is the only cause of malaria infection [25]. In this study, we characterise acquired cell-mediated immunity by following *P. vivax* infection.

## Materials and Methods

### 2.1 Study Populations

This study was approved by the Ethical Approval Committee of the Biomedical Institute of Anhui Medical University. Written informed consent was obtained from each individual before a blood sample was taken.

Blood samples were collected from 37 patients with acute *P. vivax* infections (AC) at Wuhe County Hospital, Guzhen County Hospital, and The First Hospital of Bengbu in Anhui Province in China. The patients were enrolled sequentially during June and July of 2009 and 2010. All patients enrolled in this study are inhabitants of Wuhe County, Guzhen County or the Bengbu City suburbs. Malaria transmission in this region is non-stable but can lead to malaria endemic in China. In the 1960s to 1970s, there were two malaria epidemics which were primarily caused by the *P. vivax* parasite. *P. falciparum* and *P. vivax* parasites were found together in this region until the end of the 1980s, but *P. falciparum* has not been found since the early 1990s. During the first decade of this century (from 2000 to 2010), malaria in this and other regions of China was mainly caused by the *P. vivax* parasite.

Diagnosis of *P. vivax* malaria infection was based on the examination of Giemsa-stained thick blood films. To prevent the misdiagnosis of mixed infection, confirmation of *P. vivax* was performed by polymerase chain reaction (PCR) using species-specific primers [26]. Clinical characteristics of the subjects are listed in Table 1. None of the volunteers were treated with a radical cure before blood collection. After collection, all malaria patients were cured by the standard regimen recommended by the Chinese Ministry of Health [27]. All patients were asked to follow up after blood collection; however, only 10 patients consented to give the follow up blood samples on days 7 and 14.

**Table 1:** Information 0061nd clinical data for *P. vivax* patients, uninfected malaria-exposed patients, and unexposed controls.*

	N	Sex		Age	Parasitaemia	Temperature
		M	F	years (range)	(%)	(^o^C)
*P. vivax* infection	37	22	15	29 (19–47)	0.15 (0.05–0.50)	38.5 (37.8–39.0)
Uninfected malaria-exposed controls	17	11	6	33 (19–49)	0	36.5 (36.0–37.0)
Unexposed control	21	10	11	29 (23–41)	0	36.5 (36.0–37.0)

* mean (range)

Blood samples were collected from 17 additional healthy volunteers who were randomly selected from the same *P. vivax*-endemic area during the same period of time. These subjects, serving as uninfected malaria-exposed controls (UM), had *P. vivax* infections in the twelve months prior to the time of blood collection. Negative *P. vivax* parasitaemia was confirmed by PCR with species-specific primers for four strains of human malaria (*P. falciparum, P. vivax, P. malariae, and P. ovale*) [26]. Twenty-one healthy adults living in the Bengbu city area without previous malaria infection and with no antibodies to malaria parasites, as determined by ELISA, were recruited to serve as unexposed controls (UC).

### 2.2 Preparation of Peripheral Blood Mononuclear Cells (PBMCs)

Venous blood samples from *P. vivax* patients, uninfected malaria-exposed controls, and unexposed controls were collected in heparinised tubes, and PBMCs were freshly separated by gradient centrifugation using Ficoll-Hypaque lymphocyte isolation kits (Tian Ji Hao Yao Biological Manufacturer, Tianjing, China) according to the manufacturer’s recommendations. The PBMC pellet was resuspended at 10^6^ cells/mL in RPMI-1640 (GIBCO, Carlsbad, USA) supplemented with 10% heat-inactivated fetal calf serum (FCS) (Hangzhou Sijiqing Organism Engineering Materials, Hangzhou, China). The PBMCs were counted and resuspended in the RPMI-1640 for further experiments.

### 2.3 Parasite Cultures and Antigen Preparations

*P. vivax*-infected red blood cells (iRBC) enriched from the blood of acute *P. vivax*-infected Chinese patients were used as antigens for *in vitro* stimulation. Briefly, white blood cells were depleted from *P. vivax* infected blood by filtering through a sterile CF11 cellulose column (Whatman®, Maidstone, UK). Red blood cells were washed with RPMI-1640 by centrifugation at 1,190 *g* for 5 min. The parasites were cultured for 24–30 hrs at 5% hematocrit in McCoy’s 5A medium (GIBCO) supplemented with 25% human AB serum [4]. *P. vivax* from all samples was pooled to gain a large number of parasite antigens. For *P. falciparum*, the infected blood from acute *P. falciparum*-infected Thai patients was collected, white blood cells were removed, and the parasites were cultured at 5% hematocrit in RPMI-1640 medium supplemented with 10% human serum [28]. The isolated *P. falciparum* parasites from all samples were pooled to recover a large number of parasite antigens. Both *P. vivax* and *P. falciparum* parasites were maintained in an incubator containing 5% CO_2_, 5% O_2,_ and 90% N_2_ until the parasites matured to schizont stages (≥6 nuclei). The late stage iRBCs were enriched by gradient centrifugation using 60% Percoll (GE Healthcare, Uppsala, Sweden) at 1,190 *g* for 10 min. The range of parasite purity was 60–99% for *P. vivax* and 80–100% for *P. falciparum*.

The enriched iRBC pellets were sonicated for 40 sec at 150 watts, and the protein concentration was determined by Bradford assay (Bio-Rad, Hercules, USA). The proteins were then aliquoted and stored at −70°C until use. Uninfected RBCs were processed as above, and the RBCs with a protein concentration equal to the malaria antigens were stored at −70°C to be used as a control.

### 2.4 Determination of Anti-*P. vivax* or anti-*P. falciparum* antibodies by Enzyme-linked Immunosorbent Assay (ELISA)

To determine the level of anti-*P. vivax* or anti-*P. falciparum* antibodies in *P. vivax*-infected patients, uninfected malaria-exposed and unexposed control groups, we performed an ELISA as previously reported [21]. Briefly, *P. vivax, P. falciparum* antigens, or uninfected red blood cell lysates (10 µg/ml) were coated into a 96-well plate. Plasma, at 1∶100 dilutions, was added into duplicate wells and incubated for 2 hrs at 37°C. Horseradish peroxidase-conjugated goat anti-human IgG (Caltag, Burlingame, USA) was added and incubated for 1 hr at room temperature, and 2, 20-azino-di- (3-ethylbenzthiazoline sulphonic acid) containing 50% hydrogen peroxide (Kirkepaard & Perry Laboratories, Gaithersberg, USA) was added and incubated for 30–60 min in the dark at room temperature. Enzyme activity was measured by an automated microplate reader. All values were subtracted from the baseline value obtained using uninfected red blood cell lysate before performing data analyses.

### 2.5 *In vitro* Stimulation

PBMCs from ten uninfected malaria-exposed controls, ten unexposed controls and ten *P. vivax* patients were freshly isolated and used for *in vitro* stimulation. PBMCs in RPMI-1640 supplemented with 25 mM HEPES, 2 mM glutamine, 40 µg/ml gentamicin and 10% heat-inactivated FCS were cultured at 2×10^5^ cells/well in a round-bottom, 96-well plate (Costar, Corning, USA) for 5 days at 37°C and 5% CO_2_ in the presence of 1, 10, or 50 µg/ml of either the *P. vivax* or *P. falciparum* antigen (AMB47). An equivalent concentration of proteins from nRBC or medium alone was used as baseline stimulation for the lymphocytes. All stimulation values were subtracted from the median percentages of the baseline values (calculated by either values from medium alone or unstimulated RBCs). An anti-CD3 OKT antibody (2 µg/ml) was used as the positive control. All samples were performed in triplicate. After five days of activation, the cells were pulsed with 1 µCi H^3^-Thymidine for 16–18 hrs. The proliferation of PBMCs was then determined by β-counter FJ-353 (262 Factory, Xian, China), and the data are represented as counts per minute (CPM). The CPM values from the stimulation assay were calculated from the CPM of antigen-stimulated cultures divided by the values from cultures without antigen stimulation.

### 2.6 Determination of Surface and Intracellular Proteins by Flow Cytometric (FCM) Analysis

Two hundred microliters (µl) of whole blood were collected. RBC were lysed with RBC lysing solution at room temperature for 15 min and the remaining cells were washed with PBS before staining using various combinations of fluorochrome-conjugated mAbs (as shown in Table 2) for 30 min at 4°C. The cell pellets were analysed on a FACSCalibur using CELLQuest software (Becton Dickinson, San Jose, USA).

**Table 2:** List of the combination of fluorochrome-conjugated anti-human mAbs used for FCM analysis.

Cell subpopulation	Combination of fluorochrome-conjugated mAbs			
	FITC	RPE	RPE-Cy5	APC
CD4+ MemoryT cells	anti-CD45RO^a^	anti-CD4^a^		anti-CD3^a^
CD8+ MemoryT Cells	anti-CD45RO^a^		anti-CD8^a^	anti-CD3^a^
Gamma deltaT cells	anti-gamma9^b^		anti-CD3^a^	
B cells		anti-CD19^a^	anti-CD3^a^	
Regulatory T cells	anti-Foxp3^c^	anti-CD25^b^	anti-CD3^a^	anti-CD4^a^
NK and NK T cells	anti-CD56^a^		anti-CD3^a^	

a Caltag, Burlingame, USA;

b Immunotech, Marseille, France.

c This mAb was conjugated with Alexa fluor® 488 (BioLegend, San Diego, USA).

For intracellular staining, the cells were washed with a permeabilising solution according to the manufacturer’s instructions (BioLegend, San Diego, USA). The cells were incubated in the permeabilising buffer for 20 minutes at room temperature. Alexa fluor® 488-labeled anti-Foxp3 (BioLegend, San Diego, USA) was added and incubated for 30 min at room temperature. The cells were washed with PBS and fixed with 2% paraformaldehyde in PBS for acquisition and analysis on the FACSCalibur.

See the Supplementary Figure S1 for the FACS analysis of CD4^+^CD25^hi^FOXP3^+^ T cell populations.

### 2.7 Data Analysis

**Figure 1:**
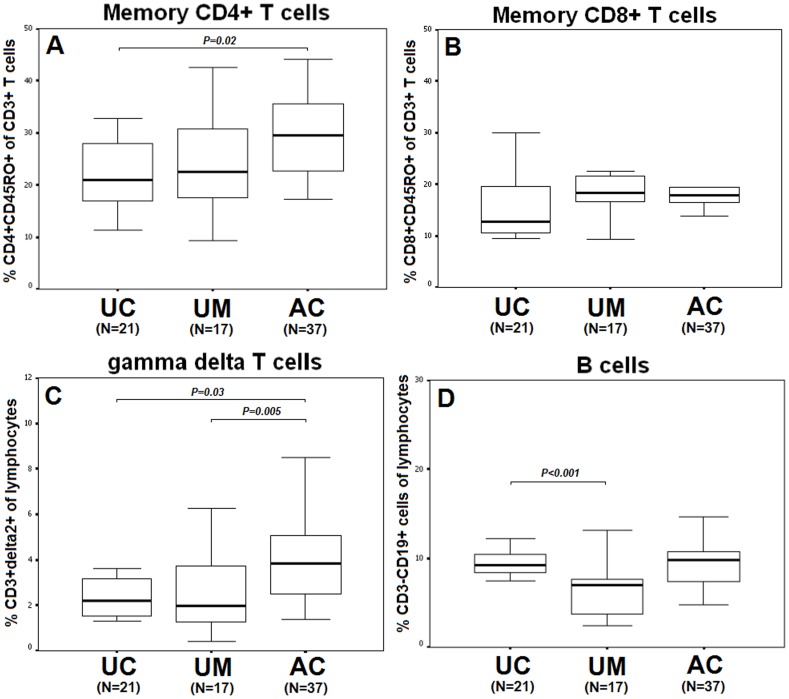
Comparison of memory CD4^+^ T cell (A) and CD8^+^ T cell (B), gamma delta T cell (C) and B cell (D) phenotypes between unexposed controls (UC:N = 21), uninfected malaria-exposed subjects (UM: N = 17), and patients with acute *P. vivax* infection (AC:N = 37). Data are represented as median, inter-quartile ranges (box plots) and maximum and minimum (upper-lower lines).

**Figure 2:**
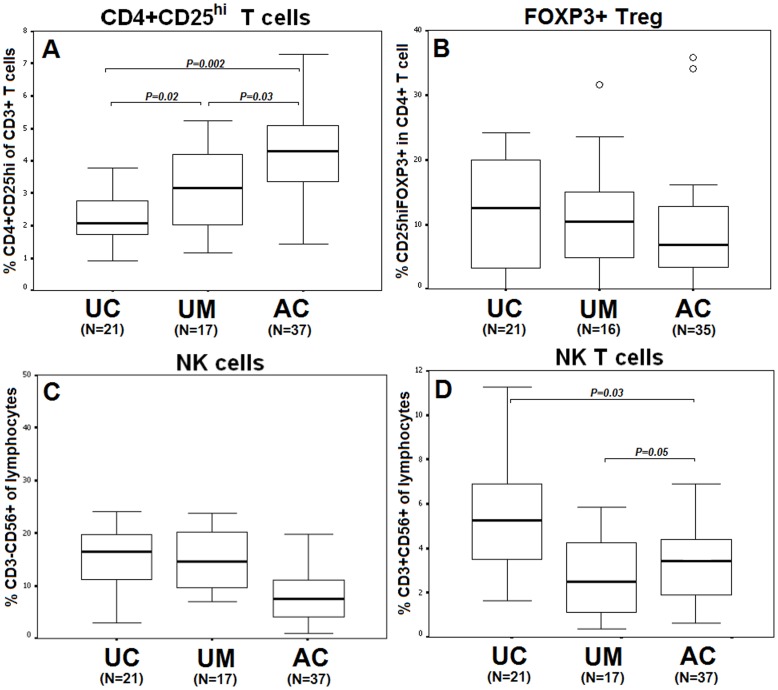
Comparison of regulatory T cell (CD4^+^CD25^hi^) (A) and CD4^+^CD25^hi^FOXP3^+^ (B), NK cell (C), and NKT cell (D) phenotypes between unexposed controls (UC:N = 21), uninfected malaria-exposed subjects (UM: N = 17), and patients with acute *P. vivax* infection (AC:N = 37). Data are represented as median, inter-quartile ranges (box plots) and maximum and minimum (upper-lower lines).

**Figure 3:**
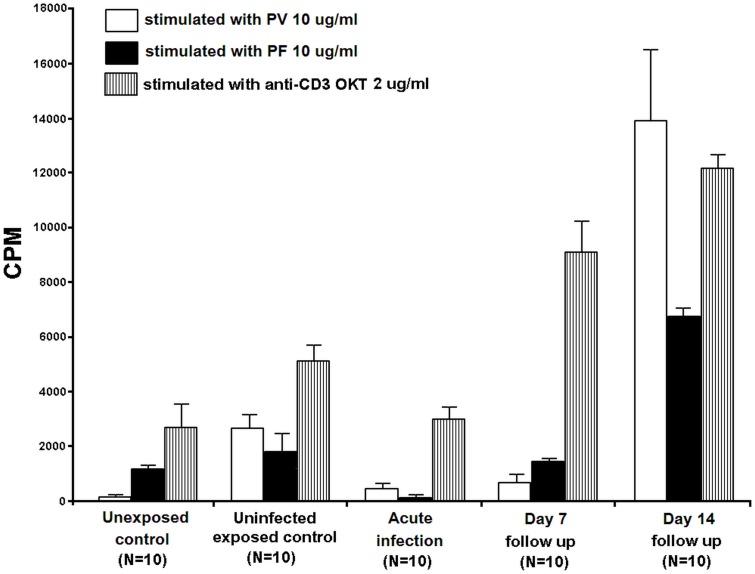
The activation of PBMCs from unexposed controls (N = 10), uninfected malaria-exposed controls (N = 10), and acute *P. vivax*-infected patients (N = 10), which was followed up on day 7 (N = 10) and day 14 (N = 10) with *P. vivax* antigens (10 µg/ml) (PV10) and *P. falciparum* antigens (10 µg/ml) (PF10). The proliferation values were expressed as counts per minute (CPM). Data are shown in median±SE. All stimulation values were subtracted from the median percentages of baseline value.

All data were analysed using the SPSS programme (Version 11.5, Chicago, USA). Non-parametric Kruskal-Wallis tests, followed by post tests, were used for statistical comparisons among three groups (Fig. 1 and 2). The data from phenotypic analyses of unstimulated lymphocytes in the results and figures (Fig. 1 and 2) were expressed as medians and interquartile ranges (25^th^–75^th^ percentile). The mean differences (MD), 95% confidence intervals (CI), and *P-values* from the statistical analyses are also represented. The results were considered statistically significant when *P<0.05* at a 95% confidence interval. The data from the *in vitro* studies (Fig. 3) are represented as the median ± standard error of the mean (SEM).

## Results

### Phenotypic Analysis of Unstimulated Lymphocytes

Acute *P. vivax* infection modulated immune function by altering the reciprocal ratios of the major immune cells in various lymphocyte populations. The percentage of CD4^+^CD45RO^+^ memory T cells out of the total number of T cells (27%, N = 37, Fig. 1A) in the acute infection was significantly higher than that of the unexposed controls (20%, N = 21, MD = 8.8, 95%CI = 0.5–17.1, *P = 0.02*). However, this result was not significant when compared with the data from the uninfected malaria-exposed controls (22%, N = 17, *P = 0.1*). In contrast, the level of CD8^+^CD45RO^+^ memory T cells among patients with acute *P. vivax* infection (17%, N = 37) was similar to that of the uninfected malaria-exposed controls (18%, N = 17). These levels were higher than, but not significantly different from, the levels observed for the unexposed controls (13%, N = 21, *P = 0.2*, Fig. 1B).

The median percentage of CD3^+^δ2^+^ γδ T cells out of the total number of lymphocytes was significantly higher in the acute *P. vivax* infection (4%, N = 37) compared to the unexposed controls (2%, N = 21, MD = 2.1, 95%CI = −0.1–4.2, *P = 0.03*) and the uninfected malaria-exposed controls (2%, N = 17, MD = 3.2, 95%CI = 1.0–5.4, *P = 0.005*, Fig. 1C).

The median percentage of CD3^−^CD19^+^ B cells out of the total number of lymphocytes was significantly lower in the uninfected malaria-exposed group (6%, N = 17) compared to that of the unexposed controls (9%, N = 21, MD = 3.5, 95%CI = 0.9–6.0, *P<0.001*). During acute *P. vivax* infection (N = 37), the percentage of B cells was unchanged, showing similar levels to the percentages observed for unexposed controls (9%, N = 21, Fig. 1D).

Among CD3^+^ T cells, the median percentage of CD4^+^CD25^hi^ T cells (Fig. 2A) in the uninfected malaria-exposed controls was significantly higher (3%, N = 17) than that of the unexposed controls (2%, N = 21, MD = 0.9, 95%CI = 0.1–1.7, *P = 0.02*). Moreover, the percentage was significantly elevated due to acute *P. vivax* infection (4%, N = 37) compared with the percentage observed in the unexposed controls (2%, N = 21, MD = 2.0, 95%CI = 0.7–3.0, *P = 0.002*) and the uninfected malaria-exposed controls (3%, N = 17, MD = 1.3, 95%CI = 0.9–2.7, *P = 0.03*).

In contrast to the percentage observed in the CD4^+^CD25^hi^ T cells, the median percentage of Foxp3^+^ Treg (CD4^+^CD25^hi^FOXP3^+^ T cells) for the total number of CD4^+^ T cells among the unexposed controls was high (14%, N = 21) but not significant when compared with the percentage observed for the acute infection group (7%, N = 35, *P = 0.2*) and the uninfected malaria-exposed controls (10%, N = 16, *P = 0.1*). Two out of thirty-seven of the acute *P. vivax* infected patients had 35% FOXP3^+^ Treg (*P = 0.001,* compared to *P. vivax*-infected patients), and 1 out of 17 of the uninfected malaria-exposed controls had an extraordinarily high level (31%) of FOXP3^+^ Treg (*P = 0.001,* compared with the uninfected malaria-exposed control group, Fig. 2B).

The median percentage of NK cells in total lymphocytes decreased but was not significant during the acute *P. vivax* infection (6%, N = 37) compared with the results observed for the uninfected malaria-exposed controls (15%, N = 17) and the unexposed group (16%, N = 26, *P>0.05*, Fig. 2C). In contrast, the percentage of NKT cells out of total number of lymphocytes (Fig. 2D) was significantly higher during acute infection (3%, N = 37) compared with the uninfected malaria-exposed group (2%, N = 17, MD = 1.4, 95%CI =  −0.5–3.0, *P = 0.05*) and was significantly lower than the results observed for the unexposed control group (5%, N = 21, MD = 1.4, 95%CI =  −0.2–3.0, *P = 0.03*).

### Proliferation of T Lymphocytes by Cross Protein Stimulation

Optimisation of PBMC stimulation was performed with PBMCs from 10 malaria patients using various concentrations of *P. vivax*, *P. falciparum,* and nRBC antigens. Stimulation with 10 µg/ml of *P. vivax* or *P. falciparum* antigens substantially elevated the level of H^3^-Thymidine uptake compared to cultures without antigen (data not shown). Therefore, further PBMC activation assays were performed using 10 µg/ml of malaria protein.

PBMCs from 10 healthy donors (unexposed) were co-cultured with the nRBC, *P. vivax,* and *P. falciparum* antigens to determine the baseline activation as shown in Fig 3. The CPM values showed a low-level response to *P. vivax* antigens (median±SEM = 152±80 CPM). Higher proliferation was observed when PBMCs were activated with the *P. falciparum* antigen (1186±247 CPM) compared to their activation by anti-OKT (2800±923 CPM).

PBMCs from 10 uninfected malaria-exposed controls co-cultured with nRBC, *P. vivax,* and *P. falciparum* antigens showed a more robust response to *P. vivax* antigens (2951±452 CPM) than *P. falciparum* antigen (1963±591 CPM) compared to the activation of PBMCs that was observed using anti-OKT (4968±606 CPM).

PBMCs from 10 acute *P. vivax*-infected patients and from their follow-up periods on days 7 and 14 were co-cultured with the nRBC, *P. vivax,* and *P. falciparum* antigens to determine the cross stimulation between the two malaria species. PBMCs from acute *P. vivax*-infected patients stimulated with *P. vivax* antigen showed a higher degree of proliferation (464±417 CPM) compared to PBMC stimulation with the *P. falciparum* antigen (116±110 CPM) after subtracting the background measured in the nRBC stimulated culture. Higher cell levels of proliferation were observed upon activation by anti-OKT (2916±552 CPM). The 7-day follow up PBMC proliferation assays from these patients showed no significant changes in proliferation upon stimulation with all three proteins. The stimulation by anti-OKT was 9008±1216 CPM. Interestingly, the 14-day follow up PBMC count from the infected patient group showed markedly increased proliferation upon stimulation with *P. vivax* antigens (13920±4623 CPM) compared to stimulation with *P. falciparum* antigens (6772±501 CPM, *P = 0.03*, Fig. 3). The level of stimulation induced by anti-OKT was 12209±450 CPM.

## Discussion

People in northern Anhui Province in Central China have had experience with only *P. vivax* infection. The *P. falciparum* parasite is absent in this area. Therefore, the existing immunity to malaria infection is shaped exclusively based on exposure to the *P. vivax* parasites. The T cell phenotypes in people infected with only *P. vivax* have not been previously investigated. Our major finding was that upon acute *P. vivax* infection, CD4^+^ memory T cells and γδ T cells were elevated in the blood. CD8^+^ memory T cells were only detectable in the acute-infected and uninfected malaria-exposed patients. Moreover, a non-immune suppressive phenotype was found in *P. vivax* infection, demonstrated by the decreased expression of FOXP3^+^ Treg cells.

Elevation of CD4^+^ memory T cells in acute *P. vivax*-infected patients in China was consistent with a study in Thailand that showed that during both acute infection and the convalescent period, similarly high levels of CD4^+^ memory T cells were present [21]. However, the levels of both CD4^+^ and CD8^+^ memory T cells in acute *P. vivax* patients from Thailand were higher than the levels of these cells observed in China. The high level of CD4^+^ and CD8^+^ memory T cells may be due to frequent exposure to malaria parasites among the Thai population. A previous study has shown IL-4-producing CD4^+^CD45RO^+^ T cells in the circulation of people who are exposed to attenuated *P. falciparum* sporozoites [29]. This finding has been confirmed by recent studies showing the presence of IL-4-producing CD4^+^ memory T cells (CD45RO^+^CD27^−^) induced by attenuated *P. falciparum* sporozoites. [21], [30]. These memory T cells may also confer protection against *P. vivax* and long-term protection against *P. falciparum* infection. However, a recent study has demonstrated that T cells from naïve individuals (without previous exposure to malaria) can differentiate into memory T cells when exposed to cross-reacting malaria antigens. Therefore, the high level of memory T cells observed during acute *P. vivax* infection suggests memory T cell activation by the parasites or priming by exposure to commensal micro-organisms, pathogens and/or vaccine antigens carrying minimal T cell epitopes that cross-react with malaria proteins [31] . In comparison to the healthy group, the level of CD8^+^ memory T cells was readily detectable in *P. vivax*-infected patients. This finding has been confirmed by recent studies showing enhanced effector T-cell populations during acute *P. vivax* infection [32]. However, cytotoxic T cells have been shown to play a minor role in murine malaria, especially in the pre-erythrocytic stage [33]. Therefore, it is expected that regular exposure to *P. vivax* parasites could maintain cytotoxic T cells in uninfected malaria-exposed controls. Additionally, exposure to liver-stage antigens of *P. vivax* during an exo-erythrocytic or a hypnozoite stage could result in the generation of long-lived CD8^+^ memory T cells in immune individuals.

Elevated γδ T cells have been demonstrated in the current study and in various models of plasmodia infections [21], [34], [35], [36]. A recent study showed that γδ T cells reduce the severity of malaria in humans [13] and control the chronic parasitaemia of *P. chabaudi* infection in mice [37]. The cytotoxic role of γδ T cells was shown *in vitro* using *P. falciparum* [38], [39], [40], [41]. This result suggests that γδ T cells play both cytotoxic and regulatory roles, which presumably lead to protection. However, the role of γδ T cells in protection related to symptom intensity or parasitaemia during malaria infection needs to be investigated further.

Previous findings show the inhibition of B cell phenotypes in both *P. falciparum* and *P. vivax* infections [24], [42], [43]. In addition, our results showed the level of B cells in acute *P. vivax*-infected patients was unchanged. One possible explanation is that Treg may directly suppress B cells [44] or the proliferation of Th cells, resulting in the down-regulation of IL-2 or IL-4 production by responder lymphocytes and the subsequent suppression of B cell differentiation into plasma cells. In addition, we found significantly decreased levels of B cells in *P. vivax*-uninfected malaria-exposed controls, which was consistent with the low antibody response in *P. vivax* infection as previously shown [21]. A recent study in *P. falciparum*-exposed populations of Africa and South America showed significant changes in B cell subsets [42], [45], [46], [47]. The difference in these findings could be consistent with the conventional understanding that *P. falciparum* is a polyclonal B cell activator that stimulates the production of large amounts of antibodies in the infected host during the convalescent period. However, during *P. vivax* infection, the patients produce lower levels of antibodies compared to the patients infected by *P. falciparum* [48].

Treg constitutively express CD25, which is the IL-2/a chain receptor. Co-expression of CD25 with forkhead box protein P3 (FOXP3) dictates the immune-suppressive role of Treg via the release of IL-10 and TGF-β [49], [50]. The unchanged levels of FOXP3^+^ Treg cells among *P. vivax*-infected Chinese populations was in contrast to levels found in *P. vivax*-infected Thai and Brazilian populations, whose levels of FOXP3^+^ Treg cells were elevated [20], [51], [52]. One reason for this difference could be that patients living in the endemic areas of Thailand are likely to have experienced both *P. vivax* and *P. falciparum* infection due to the equal incidence of both species, whereas *P. vivax* is the only malaria species found in central China. Another reason could be that *P. falciparum* activates FOXP3^+^ Treg cells, as shown previously [53]. Taken together, these findings suggest that an activator for FOXP3^+^ Treg cells in the Thai patients may originate from *P. falciparum* parasites. However, we found some cases of *P. vivax*-infected patients and uninfected malaria-exposed controls that had high levels of FOXP3^+^ Treg cells. This result may be caused by frequent exposure to *P. vivax* infection among people living in endemic areas.

The reduction of NK cells in *P. vivax*-infected Chinese populations compared to healthy volunteers was consistent with what was found in *P. vivax*-infected Thai [54] and Ethiopian populations [24]. The activation of NK cells during acute infection occurred in the host and was not due to geographical region, genetic differences, or level of malaria endemicity. The protective role of NK cells in malaria infection is supported by the finding that these cells destroy *P. falciparum*-infected erythrocytes via direct contact [34], [55]. Moreover, a recent study showed that NK cells in peripheral blood are the primary producers of IFN-γ upon exposure to the infected erythrocytes in the presence of IL-12 [34]. Therefore, NK cells may be the prime effectors against *P. vivax* parasites during the acute phase of infection, although only a small change in the level of NK cells was observed. However, the cumulative number of these cells present during the entire course of infection could not be calculated. Therefore, the suppressive effect of Treg and the early activation of NKT cells towards the *P. vivax*-infected erythrocytes were exerted at the onset of the infection. However, the lower number of NK and NKT cells found during the acute infection period compared to the number of cells observed in the malaria-unexposed controls may be explained by the incidence of lymphopoenia documented during both *P. falciparum* and *P. vivax* infections [24], [56], [57].

The results of stimulation of acute *P. vivax*-infected patients during the 7-day follow up were similar to those of the unexposed controls, suggesting drug resistance or immune suppression caused by the parasites. However, all patients were given chloroquine for 3 days plus primaquine for 8 days for treatment of *P. vivax*-induced malaria. Several studies have investigated the effect of chloroquine on the human immune system *in vivo,* with a particular focus on immune suppression [58], [59]; however, the mechanisms underlying this treatment are not thoroughly understood. In our experiments, PBMCs from all day 7 follow up patients were collected 4 days after chloroquine treatment was completed. This finding suggests that there were residual effects of chloroquine on the T cells. However, the response of the patients’ lymphocytes during the convalescent period in this study was not restricted to only *P. vivax* antigens, although *P. vivax* is the only cause of malaria infection in this area of central China.

The response of PBMCs from uninfected malaria-exposed controls to *P. vivax* and *P. falciparum* antigens suggested that parasites stimulated memory cell pools of *P. vivax*-specific lymphocytes during infection [21]. The stimulation of uninfected malaria-exposed controls and day 14 follow up PBMCs with *P. falciparum* antigens also resulted in greater levels of lymphocyte proliferation. This finding suggests antigenic cross-reactivity between *P. vivax* and *P. falciparum*, as recently suggested, by using the serological cross-reaction [13], [23] and the accumulation of both *P. vivax* and *P. falciparum*-specific memory T cells during the convalescent period, resulting in the robust response to both *P. vivax* and *P. falciparum* antigens. This finding sheds light on a possible new strategy for malaria vaccine design, i.e., a combination of antigens derived from *P. vivax* and *P. falciparum* parasites, which may attenuate malaria severity. *P. vivax* parasites do not suppress the immune function of the infected host, which commonly accompanies *P. falciparum* infection. However, elucidation of the immune cell-parasite interaction during malaria infection using single or mixed species needs to be investigated in depth to gain a better understanding of immunity to malaria infections.

## Supporting Information

Figure S1 FACS analysis of the CD4^+^CD25^hi^FOXP3^+^ T cell population (**A**) represents the CD3^+^ population. The R1 gate was used for (**B**), and R2 was the gating population of (**C**). 20000 of total evens were used for each gate.
(DOCX)
