# Supplementary material for: Immunity to Malaria in Plasmodium vivax Infection: A Study in Central China
Source: PLoS One. 2012 Sep 25;7(9):e45971. doi: 10.1371/journal.pone.0045971 (PMC3457974; doi:10.1371/journal.pone.0045971)
Supplement: Figure S1 — FACS analysis of the CD4+CD25hiFOXP3+ T cell population (A) represents the CD3+ population. The R1 gate was used for (B), and R2 was the gating population of (C). 20000 of total evens were used for each gate. (DOCX) [file pone.0045971.s001.docx]

**Supporting information**

**
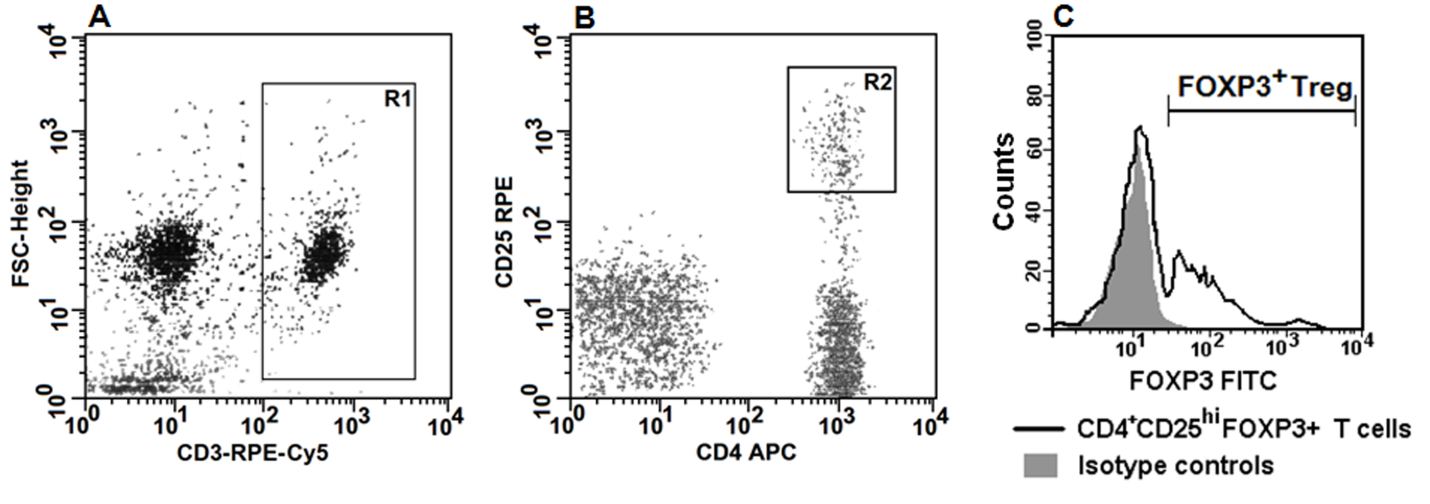
**

**Supplementary Figure S1**

FACS analysis of the CD4^+^CD25^hi^FOXP3^+^ T cell population **(A)** represents the CD3^+^ population. The R1 gate was used for **(B)**, and R2 was the gating population of **(C).** 20000 of total evens were used for each gate.
